# Supplementary material for: Comparative genomics provides new insights into the diversity, physiology, and sexuality of the only industrially exploited tremellomycete: Phaffia rhodozyma
Source: BMC Genomics. 2016 Nov 9;17:901. doi: 10.1186/s12864-016-3244-7 (PMC5103461; doi:10.1186/s12864-016-3244-7)
Supplement: Additional file 6: — List of orphan genes with links to PFAM (related to Additional file 1: Table S1). (ZIP 1428 kb) [file 12864_2016_3244_MOESM6_ESM.zip › PFAM_UNIQUE/G02767_P.html]

#                                                                            --- full sequence --- -------------- this domain -------------   hmm coord   ali coord   env coord
  
# target name        accession   tlen query name           accession   qlen   E-value  score  bias   #  of  c-Evalue  i-Evalue  score  bias  from    to  from    to  from    to  acc description of target
  
#------------------- ---------- ----- -------------------- ---------- ----- --------- ------ ----- --- --- --------- --------- ------ ----- ----- ----- ----- ----- ----- ----- ---- ---------------------
  
G02767\_P             -           1031 Adaptin\_binding      PF10199.4    137      0.22   10.5  13.5   1   1    0.0024      0.69    8.9  13.5    38   104   362   457   318   474 0.58 - Alpha and gamma adaptin binding protein p34  
G02767\_P             -           1031 Astro\_capsid         PF03115.9    787       2.6    4.8  12.2   1   1    0.0014       4.1    4.2  12.2   642   708   393   461   320   480 0.76 - Astrovirus capsid protein precursor  
G02767\_P             -           1031 CDC27                PF09507.5    430       0.5    8.2  21.4   1   1   7.7e-05     0.012   13.5  14.7   157   308   344   468   307   521 0.45 - DNA polymerase subunit Cdc27  
G02767\_P             -           1031 CDC45                PF02724.9    622      0.16    8.6  20.7   1   1     0.003      0.27    7.8  20.7   117   174   399   463   387   573 0.65 - CDC45-like protein  
G02767\_P             -           1031 Cnd2                 PF05786.9    725       7.2    3.6  15.5   1   1     0.017        15    2.6  15.5   292   371   398   474   349   501 0.66 - Condensin complex subunit 2  
G02767\_P             -           1031 DUF1510              PF07423.6    217      0.47    8.4  21.2   1   1    0.0057      0.95    7.5  21.2    50   117   401   469   380   516 0.51 - Protein of unknown function (DUF1510)  
G02767\_P             -           1031 DUF2146              PF10220.4    895      0.34    7.5   7.7   1   1   0.00018      0.55    6.8   7.7   529   621   376   465   368   511 0.58 - Uncharacterized conserved protein (DUF2146)  
G02767\_P             -           1031 Nop14                PF04147.7    840       1.4    5.4  36.9   1   1     0.039       2.6    4.5  36.9   315   391   407   464   388   552 0.55 - Nop14-like family  
G02767\_P             -           1031 PBP\_sp32             PF07222.7    243       1.1    7.0  10.4   1   1     0.003         3    5.6  10.4   139   239   362   464   345   468 0.64 - Proacrosin binding protein sp32  
G02767\_P             -           1031 PPP4R2               PF09184.6    288      0.18   10.0  26.6   1   1    0.0014      0.48    8.6  26.6   214   285   399   469   354   472 0.57 - PPP4R2  
G02767\_P             -           1031 SGT1                 PF07093.6    589       7.4    3.5  14.5   1   1     0.068        12    2.7  14.5   497   540   411   454   377   562 0.63 - SGT1 protein  
G02767\_P             -           1031 SpoIIIAH             PF12685.2    196       6.4    4.9  10.7   1   1     0.023        14    3.8  10.7    23    81   406   467   392   527 0.55 - SpoIIIAH-like protein  
G02767\_P             -           1031 Spore\_coat\_CotO      PF14153.1    185         5    5.2   6.8   1   1     0.046       9.7    4.2   6.8    49    98   415   464   380   553 0.59 - Spore coat protein CotO  
G02767\_P             -           1031 TAP42                PF04177.7    340         6    4.4   8.7   1   1     0.011        11    3.5   8.7   298   334   417   454   313   463 0.80 - TAP42-like family  
G02767\_P             -           1031 Utp14                PF04615.8    735      0.98    6.2  16.9   1   1     0.017       1.6    5.4  16.9   311   376   396   465   384   552 0.64 - Utp14 protein  
G02767\_P             -           1031 Vfa1                 PF08432.5    182       1.8    7.2  14.2   1   1     0.021       5.1    5.8  14.2    48   136   383   468   378   479 0.48 - AAA-ATPase Vps4-associated protein 1
